# Flaccid Paralysis with Hyponatremia: Think Guillain-Barre Syndrome

**DOI:** 10.7759/cureus.7113

**Published:** 2020-02-27

**Authors:** Daniel Griffin, Hussein Asad, Parth Patel, Ashraf Gohar

**Affiliations:** 1 Pulmonology and Critical Care, University of Missouri-Kansas City, Kansas City, USA; 2 Pulmonary and Critical Care, University of Missouri-Kansas City, Kansas City, USA; 3 Internal Medicine, University of Missouri-Kansas City School of Medicine, Kansas City, USA; 4 Pulmonary and Critical Care and Sleep, University of Missouri-Kansas City (Hospital Hills Campus), Kansas City, USA

**Keywords:** guillain-barre syndrome, hyponatremia

## Abstract

Guillain-Barre syndrome (GBS) is the most common cause of flaccid paralysis in affected patients. Here we present a case of GBS presenting with flaccid paralysis as well as hyponatremia. The association of hyponatremia in GBS is discussed, as well as other potential causes and risk factors.

## Introduction

Guillain-Barre syndrome (GBS) is an acute inflammatory radiculoneuropathy and is characterized by acute or subacute areflexic paralysis with albumin-cytologic dissociation evident in the cerebrospinal fluid (CSF) analysis. It is considered the most common cause of acute flaccid paralysis in regions where poliomyelitis is eradicated with an incidence of 0.8-1.9 per 100,000 people [1]. In patients with critical neurological disease, hyponatremia is one of the most severe metabolic complications. Even with mild hyponatremia, the consequences can be grave, ranging from severe neurological deficits to death [2,3]. The association between hyponatremia and GBS has been reported; however, the underlying pathophysiology is not entirely understood. This report serves to raise the awareness of GBS as a cause of severe hyponatremia and help further guide diagnostic and treatment recommendations. 

## Case presentation

A 57-year-old female presented to the emergency department due to bilateral distal extremity weakness, worse in the lower extremities, and unsteady gait. The patient stated that her symptoms progressed over the last week with significant numbness, tingling, weakness bilaterally, difficulty walking, and frequent falls. She noted that she was treated for an upper respiratory tract infection several weeks prior, with complete resolution of her symptoms and no other changes in lifestyle, vaccination, or new medications. Upon arrival, computed tomography of the head and thoracic spine was negative for acute findings. Magnetic resonance imaging of the cervical, thoracic, and lumbar spines and head was also negative for acute changes. Initial labs, including sodium, which was 136 mEq/L (reference range 132-146 mEq/L), were within normal limits. Lumbar puncture was performed, and the patient was noted to have a protein level of 116 mg/dL (reference range 15-60 mg/dL) in her CSF sample, white blood cells 2 per µL (reference range 0-5 per µL, red blood cells 6 per µL (reference range 0 per µL), and glucose 65 mg/dL (reference range 50-80 mg/dL). After an extensive workup, she was diagnosed with GBS on day 4 of her hospital stay. As this diagnosis was being confirmed, the patient’s sodium level dropped from 136 to 116 (reference range 132-146 mEq/L), urine sodium was > 40 mEq/L (reference range >20 mEq/L) and serum osmolality was 241 mOsm/kg (reference range 285-295 mOsm/kg). When assessing this time course she developed significant hyponatremia four days after hospitalization, but this was in total eight days after the onset of her symptoms. Intravenous immunoglobulin (IVIG) and hypertonic saline were initiated for the treatment of her GBS and hyponatremia. These were both done on day 4 of her hospital stay after the final diagnosis was confirmed. Over the next several days, the patient’s hyponatremia resolved with the treatment of her GBS. Her neurological symptoms substantially improved before discharge but have mildly persisted at follow-up appointments. She is currently able to ambulate without difficulty and participate in all activities of daily living.

## Discussion

The incidence of hyponatremia in GBS patients is reported to be as high as 48% and as a low of 21.5%. Studies have demonstrated that the rate of hyponatremia is more elevated in severe GBS patients than in mild-to-moderate cases. The mechanism of hyponatremia is usually attributed to the syndrome of inappropriate secretion of antidiuretic hormone (SIADH) and has been documented as an initial symptom before flaccid paralysis. Approximately 5% of hospitalized patients with GBS develop SIADH [4]. The presenting finding and frequency of GBS can be seen in Table 1. 

**Table 1:** Guillain-Barre Syndrome Presentation

Presentation	At diagnosis
Symmetrical flaccid paralysis of lower extremities	90% (10% starts in upper extremity or face)
Need for ventilatory support	10%-30%
Areflexia (at presentation)	90% (100% in progressed disease)
Pain (nerve root compression)	67%
Dysautonomia	70%
Hyponatremia	20%-40%

The association between GBS and hyponatremia is attributed to the development of SIADH secondary to hypothalamic inflammation. It has also been noted that the onset of hyponatremia in GBS has a median time of 8.8 days [5]. This is consistent with our case where she developed hyponatremia within eight days of the onset of symptoms but was four days after hospitalization. Aside from SIADH, there are additional sodium derangements that can occur in the setting of GBS. First, some patients can develop polydipsia in the context of GBS, which can lead to significant sodium abnormalities. Also, the treatment of GBS with IVIG can lead to sodium abnormalities due to several mechanisms. IVIG can cause true hyponatremia due to the movement of intracellular water to the extracellular compartment caused by sucrose in the IVIG solution. Secondly, serum sodium can be diluted by increased proteins and lipids from the IVIG solution, causing pseudohyponatremia with an elevated serum osmole level [6]. While IVIG can lead to hyponatremia, this did not cause our patient's hyponatremia as she had developed it prior to starting IVIG and in fact significantly improved with the treatment of her GBS. In our case, hyponatremia was felt to be solely due to GBS-induced SIADH. 

Risk factors for hyponatremia in the setting of GBS include age > 50 years and preexisting comorbidities, including anemia, alcohol abuse, hypertension, and coagulopathy. 

With the above-noted rates of hyponatremia and its association with more severe disease, the clinician should always closely monitor serum sodium levels in the setting of GBS, especially when treatment with IVIG is being considered. 

## Conclusions

Hyponatremia can occur in the setting of GBS, typically in association with severe clinical states. Although SIADH is the most common cause of hyponatremia in GBS, the exact underlying pathophysiology is unknown. Treatment should be directed at the underlying cause (i.e., IVIG for GBS and treatment of SIADH, accordingly).
